# Supplementary material for: From sequence to enzyme mechanism using multi-label machine learning
Source: BMC Bioinformatics. 2014 May 19;15:150. doi: 10.1186/1471-2105-15-150 (PMC4229970; doi:10.1186/1471-2105-15-150)
Supplement: Additional file 2 — Java code of ml2db. Additional file ml2db_code.tar.gz contains the Java source code to run the multi-label machine learning experiments and save the results to database. The code’s Javadoc is included. [file 1471-2105-15-150-S2.zip › additional file 2/ml2db/ml2db/doc/mulan/classifier/neural/ModelUpdateRule.html]

ModelUpdateRule


JavaScript is disabled on your browser.


- Overview
- Package
- Class
- Use
- Tree
- Deprecated
- Index
- Help

- Prev Class
- Next Class

- Frames
- No Frames

- All Classes

- Summary:
- Nested |
- Field |
- Constr |
- Method

- Detail:
- Field |
- Constr |
- Method


mulan.classifier.neural

## Interface ModelUpdateRule

- All Known Implementing Classes:
  :   MMPMaxUpdateRule, MMPRandomizedUpdateRule, MMPUniformUpdateRule, MMPUpdateRuleBase

  ---

    

  ```
  public interface ModelUpdateRule
  ```

  Represents an update rule, which can be used by a learner, to process an input
  example in learning phase and perform an update of a model when necessary.

  Version:
  :   2012.02.27

  Author:
  :   Jozef Vilcek

- - ### Method Summary

    Methods

    | Modifier and Type | Method and Description |
    | --- | --- |
    | `double` | `process(DataPair example, java.util.Map<java.lang.String,java.lang.Object> params)` Process the training example and performs a model update when suitable. |

- - ### Method Detail


    - #### process

      ```
      double process(DataPair example,
                   java.util.Map<java.lang.String,java.lang.Object> params)
      ```

      Process the training example and performs a model update when suitable.
      The decision when to perform model update is carried by the update rule
      (e.g. when the model response is not within an acceptable boundaries from
      the true output for given example).

      Parameters:
      :   `example` - the input example
      :   `params` - the additional parameters for an update.

      Returns:
      :   the error measure of the model response for given input pattern
          and specified true output.


- Overview
- Package
- Class
- Use
- Tree
- Deprecated
- Index
- Help

- Prev Class
- Next Class

- Frames
- No Frames

- All Classes

- Summary:
- Nested |
- Field |
- Constr |
- Method

- Detail:
- Field |
- Constr |
- Method
